# Ectopic Pleomorphic Adenoma of the External Nose

**DOI:** 10.1155/crot/7281902

**Published:** 2026-07-05

**Authors:** Cyrus W. Abrahamson, Julie G. Elfishawy, Lacey Durham, James C. Wang

**Affiliations:** ^1^ Feinberg School of Medicine, Northwestern University, Chicago, Illinois, USA, northwestern.edu; ^2^ Department of Pathology, Northwestern University, Chicago, Illinois, USA, northwestern.edu; ^3^ Division of Facial Plastic and Reconstructive Surgery, Department of Otolaryngology-Head and Neck Surgery, Northwestern University, Chicago, Illinois, USA, northwestern.edu

**Keywords:** minor salivary gland tumor, nasal cavity, nasal mass, pleomorphic adenoma

## Abstract

Pleomorphic adenomas (PAs) are the most common benign salivary gland tumors but rarely present in ectopic locations. When ectopic PAs occur in the head and neck, they most often arise from minor salivary gland tissue of the oral or nasal cavities. PAs of the external nose are exceedingly rare, and this is the first report of a PA arising from the nasal bridge. Here, we report a case of an African American man in his mid‐20s with no pertinent medical history who presented with a slowly enlarging left nasal bridge mass that had grown over the past two to three years. Initially attributed to prior facial trauma and managed as a keloid with steroid injections, the lesion failed to improve, prompting referral to otolaryngology. Ultrasound demonstrated a 1.1 × 1.7 × 1.2 cm heterogeneously hypoechoic subcutaneous mass, and MRI showed a T2 hyperintense, avidly enhancing mass closely associated with but not communicating with the nasolacrimal duct. En bloc excision was performed, and histopathology confirmed PA via characteristic biphasic morphology and diffuse PLAG1 positivity. This case represents the most superiorly located nasal PA in the literature, and the absence of nasolacrimal duct involvement suggests true ectopic origin rather than extension from recognized salivary elements. This case highlights the importance of maintaining a broad differential for external nasal masses, particularly when lesions fail to respond to treatment as expected.

## 1. Introduction

Masses of the nasal cavity and external nose often require excision due to impaired function or cosmetic purposes. Most lesions are benign, including developmental inclusion cysts, like dermoid or epidermoid cysts, and soft tissue tumors. However, masses can be malignant in rare cases hence the need for biopsy or excision. Additionally, there are rare instances in which ectopic tissue can present in this region, as we present here. This case report was exempt from IRB approval based on institutional policies.

## 2. Case Presentation

**FIGURE 1:**
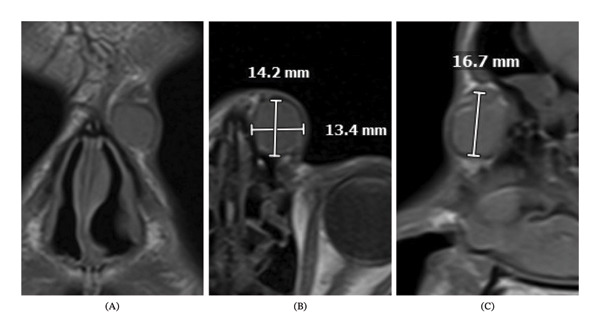
T1‐weighted MRI images: (A) coronal view, (B) axial view, and (C) sagittal view of the patient.

An African American man in his mid‐20s with no pertinent past medical history presented for a growth on his left external nasal bridge. Since he first noticed it 2‐3 years ago after sustaining a facial injury, it had been slowly growing. He denied any nasal obstruction, increased lacrimation, or rhinorrhea. He did report a family history of keloids, and it was initially thought to be a keloid prior to referral and treated with several rounds of steroid injections without improvement. Ultrasound revealed a subcutaneous 1.1 × 1.7 × 1.2 cm smooth, heterogeneously hypoechoic mass. MRI showed a 1.3 × 1.7 × 1.4 cm heterogenous mass that was T2 hyperintense and T1 isointense with avid enhancement (Figure 1A–C). The cortex of the left nasal bone was intact, and the mass was closely associated with the nasolacrimal duct, which was, otherwise, normal. There were also incidental sinonasal findings including fluid within the right frontal sinus, a tiny retention cyst in the right maxillary sinus, and mild to moderate mucosal thickening in the ethmoid air cells, none of which were felt to be clinically significant or related to the presenting mass. CT was not obtained as MRI was felt to sufficiently characterize the osseous anatomy without evidence of erosion or invasion. At this time, the patient underwent en bloc excision and the mass was sent to pathology.

**FIGURE 2:**
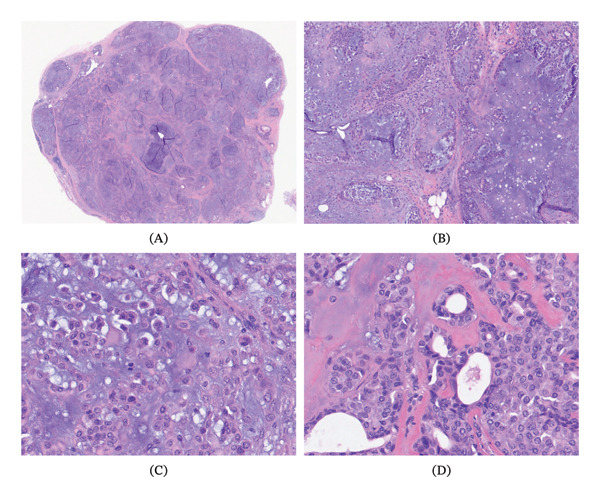
Pathology images of mass following excision: (A) low‐power view, (B) medium‐power view, and (C) and (D) high‐power view.

Pathology demonstrated a biphasic salivary gland tumor composed of ductal epithelial and myoepithelial cells embedded in a chondromyxoid stroma, pathognomonic for pleomorphic adenoma (PA). Immunohistochemistry demonstrated Ki‐67 proliferation of 10%–15% and diffusely positive PLAG1, an ancillary test serving as a surrogate marker of underlying PLAG1 rearrangement, supporting PA diagnosis. Low‐power view demonstrated a nodular mass separated by broad fibrous septa (Figure 2A). Medium‐power view demonstrated a chondromyxoid matrix with two clear cell types present (Figure 2B). High‐power view demonstrated round myoepithelial cells and multiple lumina lined by epithelial cells (Figure 2C‐D). All margins were negative.

## 3. Discussion

While this is not the first report of PA of the external nose, our literature review shows that this is the first arising from the nasal bridge and the most superiorly located nasal PA reported [1, 2]. PAs are classically tumors of the major and minor salivary glands, and ectopic presentations are rare [3]. When they do occur outside salivary tissue, they are most often found in the oral cavity within the hard and soft palates, buccal mucosa, cheek, and tongue and, less frequently in sites like the nasal septum, lateral nasal wall, thyroid, and parathyroid [4]. During embryologic development, ectopic or accessory salivary gland tissues share developmental lineage with normal salivary epithelium and can undergo the same neoplastic changes, including PA development. Additionally, normal seromucinous glands are present throughout the respiratory mucosa, so lesions in these locations may arise from normal structures. Surgical excision is the cornerstone of management for PA; if margins are positive after initial resection, re‐excision is recommended. Incomplete resection is the primary risk factor for recurrence and development of multifocal disease, with overall recurrence rates of approximately 3%‐4% [5]. Current literature suggests that ectopic presentations of PA do not confer increased risk of recurrence or malignant transformation; however, given these lesions’ rarity, continued follow‐up is advised.

This lesion’s position on the nasal bridge and lack of communication with the nasolacrimal duct make it distinct. The absence of anatomic connection suggests that this tumor represents true ectopic implantation rather than a neoplasm arising from unrecognized salivary elements. This is especially notable in a young patient because PAs of the external nose are exceedingly uncommon at any age and most often present in middle adulthood. The relatively young age adds to the rarity and reinforces the need to consider atypical lesions when a mass does not follow the expected clinical pattern. Postoperative follow‐up was limited as the patient missed scheduled appointments; however, external records demonstrated no recurrence or new nasal mass at outside institutions. Given the rarity of this presentation and the known recurrence risk of approximately 3%‐4% with negative margins, continued long‐term surveillance is advised [5].

Overall, this case underscores the importance of maintaining a broad differential diagnosis for superficial nasal masses. The most common etiologies include nasal developmental inclusion cysts and soft tissue tumors. However, unusual lesions such as PA can mimic more common entities, and clinicians should remain open to further workup or advanced imaging when the clinical course, location, or histologic features do not align fully with the expected diagnosis.

## Author Contributions

Cyrus W. Abrahamson: data curation, visualization, and writing–original draft. Julie G. Elfishawy: data curation, visualization, and writing–original draft. Lacey Durham: data curation, visualization, and writing–review and editing. James C. Wang: conceptualization, project administration, supervision, and writing–review and editing.

## Funding

The authors received no funding for this work.

## Consent

This study was deemed exempt by Northwestern University’s IRB and deidentified in accordance with ICMJE guidelines to maintain patient anonymity.

## Conflicts of Interest

The authors declare no conflicts of interest.

## Data Availability

The data that support the findings of this study are available from the corresponding author upon reasonable request.
